# Scenarios of Engineered Membranes in Cultural Heritage: How Do Membranes Act on Microclimates and Microenvironments?

**DOI:** 10.1021/acsomega.6c02101

**Published:** 2026-08-10

**Authors:** Annarosa Gugliuzza

**Affiliations:** † National Research Council of Italy (CNR) - Research Institute on Membrane Technology “Enrico Drioli”- (ITM), via Pietro Bucci 17C, Rende, Cosenza 87036, Italy; ‡ Common laboratory of Science for Cultural Heritage | CHrossLab, Department of Chemical Science and Materials Technologies (CNR-DSCTM), via dei Taurini 19, Roma 00185, Italy

## Abstract

Conservation is one of the critical issues for cultural heritage assets. A lot of materials are daily proposed to clean, repair, and restore monuments, but best practices are still in high demand to prevent artwork deterioration. Membrane science provides practical and sustainable solutions, including buffer environmental zones, to preserve cultural assets from physical, chemical, and biological agents. Protection can be obtained in an outdoor context in order to counteract the effects of climatic changes, variations of temperature, violent storms, bright lighting, and the wear of time. Removable and aesthetic structural membranes are hence proposed to give protection and a comfortable environment without affecting the meaning of the historical monuments. Similarly, indoor membrane-based solutions can be applied to versatile situations, yielding surroundings targeted for specific classes of materials. Required ambiences may be identified, and regulation of levels of humidity, gases, and vapors, as well as fluctuations of heat can be established. Air cleaning can be obtained through the elimination of nanoparticles and dust, while bacteria, fungi, molds, and viruses can be in situ neutralized. Water infiltration and salt crystallization can be hindered. The membrane concept relies on the capability to transfer mass, energy, and charge through an interface under specific working conditions and can be easily adapted to the space needing protection. Due to their ability to perceive changes in the local environment, responsive membranes can be further aimed at realizing intelligent devices if integrated with AIoT monitoring tools. Different contexts are herein analyzed and discussed, highlighting the great potential of using advanced materials-membranes to keep balanced and clean ambiences for safe and wholesome cultural assets. Case studies are reported on how new sustainable practices can make preventive action efficient, quick, and durable, resulting in much more effective and resourceful conservation. Current status, potential membrane applications, and future perspectives are herein described in order to provide answers to some of the most important questions in works of art conservation, which are still unsolved or partially solved.

## Graphical abstract

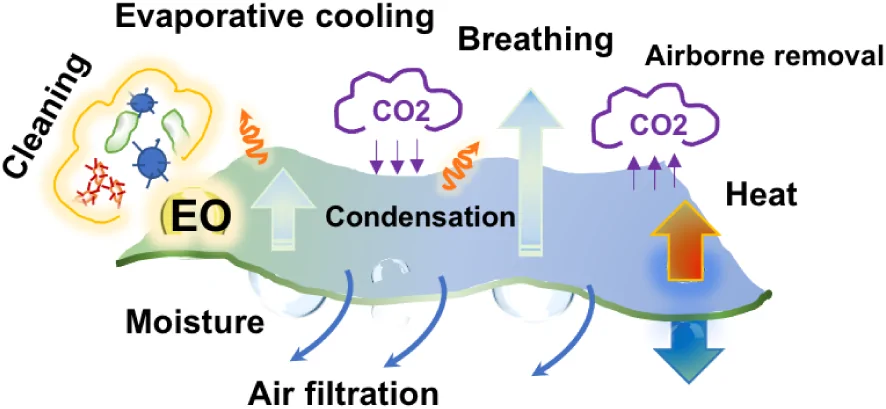

## Introduction

Membrane engineering provides eco-friendly solutions and viable practices in global and competitive contexts, including heritage conservation.^1^ Membranes can provide a buffer to the surrounding environments when working as structural elements of devices dedicated to protection against sudden environmental changes. The latter are regarded as responsible for the destabilization of equilibrium that is instead necessary to preserve the works over time.^2^ As is known, prolonged exposure to daylight, temperature and relative humidity fluctuations, increases in CO_2_, or harsh gases and vapors along with other chemical, physical, and biological pollutants are among the most frequent causes of degradation of heritage assets.^3–5^ Cracking of wood supports, tears on canvases, textiles, and leather, material loss and bloom, as well as metal oxidation, fractures, and crumbling in stones, are the result of the negative and long-term effects of external variables.^6–9^ Adaptive measures have been proposed to preserve historical buildings;^10^ however, the integrity of historical artifacts depends on surrounding climatic conditions, which are connected to territory, seasonal weather, climate changes, the number and frequency of visitors, emission densities, and distribution patterns of ambient-air pollution, as well as uniqueness of the architecture and building materials.^11,12^ Advanced monitoring actions are frequently matters of research that consist of tracking the state of cultural assets and identifying potential risks;^13,14^ however, the greatest criticality lies in collecting uniform temporal data at a rate that matches the pace at which surfaces and materials change.^15^ Regardless of how sophisticated the monitoring may be, it is however necessary to integrate it with devices capable of counteracting material (bio)­deterioration, aging, and other kinds of transformation. Membranes can shelter ambiences subjected to microclimate fluctuations, humidity stress, heat islands, infiltration, salt crystallization, biodeterioration, and corrosion. In response to the problem of degradation due to changes in the environment and human activities, neighboring buffer zones could be realized through the choice of sustainable materials and nanotechnologies, which can be removed at any time and do not leave unsafe traces on the architecture and surfaces of monuments as well as artifacts.^16^ In membranes, there is an inherent concept of sustainable dynamic interfaces, which need to be adapted to the surrounding environment by minimizing potential damage to the original structures.^1,17^ On the other hand, bio-based materials as well as bioinspired approaches are often preferred to make the membranes suitable tools for interacting safely with objects and surrounding environments. The option of using membrane-based devices for preventive conservation is, however, recent, limited to some aspects such as biodeterioration and shielding mainly and not fully exploited within the context of microclimate (Figure 1).

While the literature reports many works—more than 300—on cultural restoration and conservation over the last 30 years, only a small number of contributions having a peak in 2023 include the membrane concept, with a major effort from Italy that is a country rich in cultural gems (https://www.scopus.com). The trend in the literature highlights an increasing attention to restoration and conservation of works of art due to a shared awareness of the cultural heritage significance. The predominant topic is the assessment of issues that affect integrity, usability, and conservation, including microclimate. Regarding microclimate aspects, the discussion is mainly dedicated to discerning between cause and effect, whereas resolutive, adaptable, and scalable solutions are not yet considered fully. Monitoring provides indication about changes of variable magnitudes but does not readdress designed ambiences alongside.

**Figure 1:**
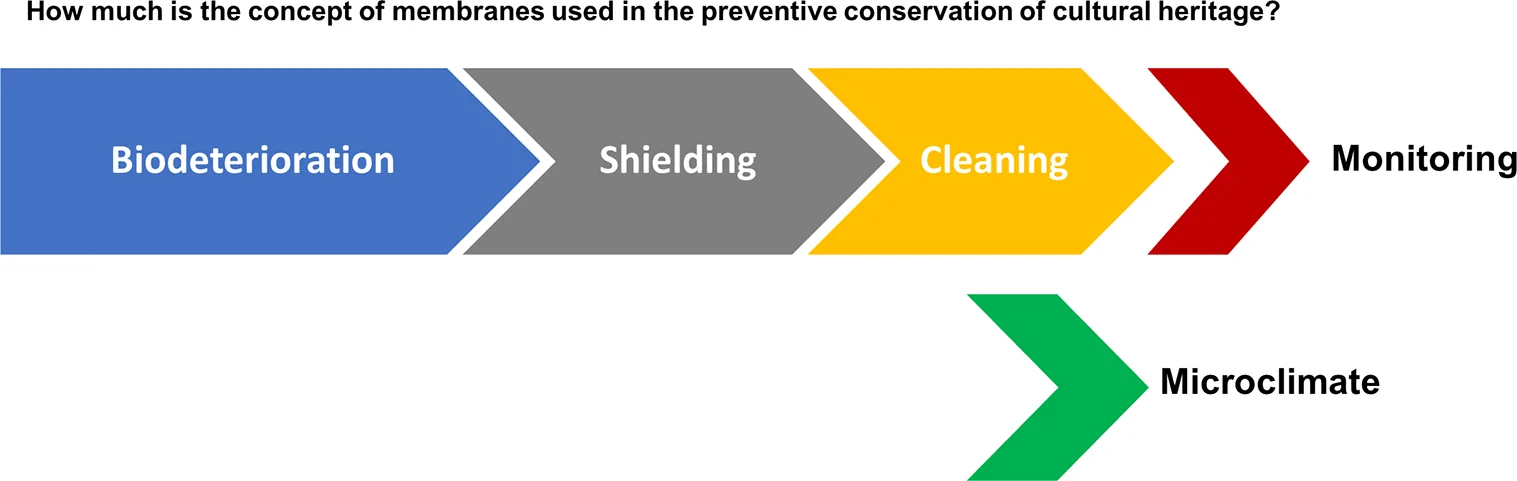
Indicative use of membrane concept in some research areas related to the preventive conservation of cultural heritage.

Herein, the potential of engineered membranes in cultural assets is examined, with a specific focus on how they may provide environmental buffer zones. This is to prevent the ambiences hosting monuments, buildings, and artworks from facing imbalance risks. State-of-the-art advancements and perspectives are therefore discussed within the context of membranes for heritage conservation.

Before entering into the merits, some basic concepts regarding membrane types and transport mechanisms are introduced. Useful elements are provided to assess how membranes meet the criteria of sustainability, which is regarded as one of the five pillars of actions surrounding cultural heritage. Precisely, the role of membranes is discussed in outdoor and indoor contexts, with special attention to how they counteract negative effects due to climate change, uncontrolled degradation, and overtourism.^18^

## Membrane Concepts and Transport Fundamentals

Membranes offer sustainable solutions as protective shelters and permselective systems in confined air. They find their natural application within the framework of microclimate and microenvironmental concepts. Because membranes can be scaled, miniaturized, and properly engineered, they are adaptable to different extents, types, and qualities of space. In this regard, it is useful to distinguish between the concept of microclimate that implies a volumetric space limited by outer walls of a building and that of the environment, regarded as an open space like a floor or a big room within a building. Likewise, what surrounds a single work is defined as a microenvironment. Therefore, to establish which kind of membrane can be beneficial to the environment, variables with the greatest impact on the healthiness of each context need to be examined.

It is relevant to observe that membranes can work as passive or interactive films depending on structure–chemistry relationships that distinguish them. It is also imperative to discern that events take place not only on the membrane surface but also in the bulk, since the membrane concept is an expression of something capable of interacting and recognizing but above all separating. Some fundamental mathematical equations are hereafter reported as useful support to understand how a membrane can be managed to provide practical solutions within the context of cultural heritage conservation. Therefore, it is permissible to ask some questions.

### Mass Transport Mechanisms through Membranes

In the most traditional meaning, the membrane is a permselective physical barrier enabling the separation of molecular species under a specific driving force, i.e., Δ*T*, Δ*p*, Δ*C*, Δ*E*, according to passive mechanisms that depend on intrinsic morphological and chemical features of the membranes (Figure 2a). Provided that the pore is the discriminant morphological element for transmembrane transport (Figure 2b), a summary of the main mechanisms, which regulate the molecular diffusion through free paths inside the membrane, is hereafter given.

**Figure 2:**
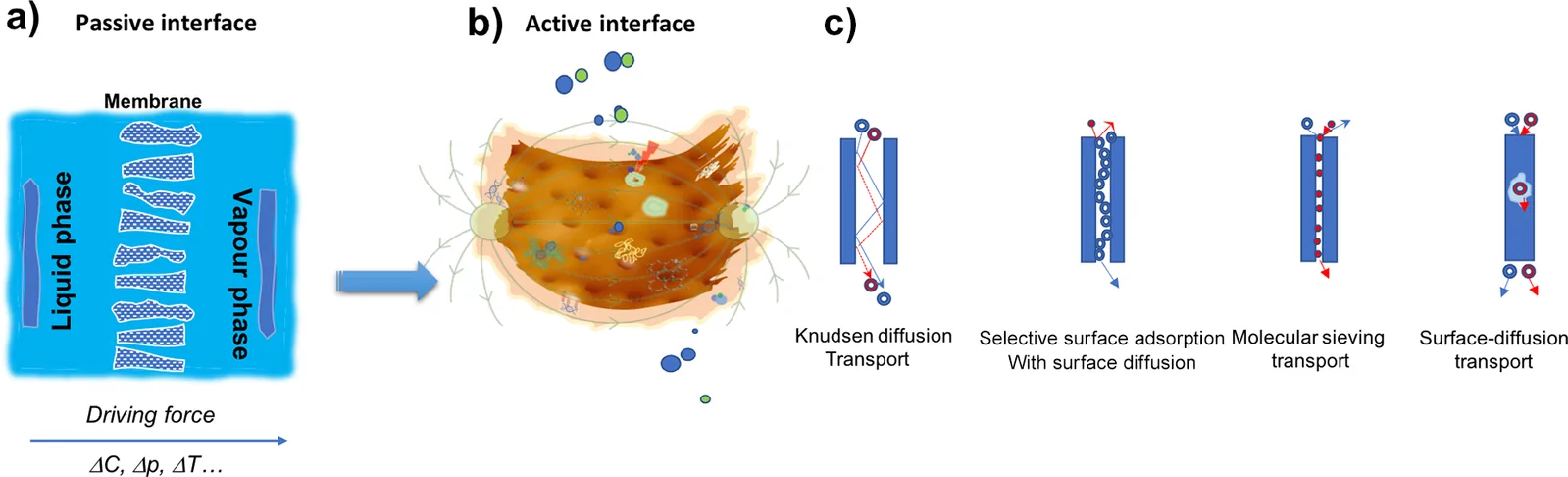
Representative scheme of passive (a) and active membranes (b) and classical transport mechanisms through membranes (c).

Depending on the pore size, the molecular motion through the membrane can take place according to convective Poiseuille flow, Knudsen diffusion, surface diffusion, capillary condensation, molecular sieving, and solution diffusion.^19^ Mathematical equations summarized in Table 1 enable the transport through membranes to be quantified as a function of intrinsic structural features of both the membrane and the diffusing molecular species.

**Table 1:** Mathematical Equations Describing Main Diffusion Mechanisms through Membranes

Mechanism	Mathematical equation	Notes
Poiseuille flow	1 $J=\frac{\epsilon \eta r^{2}}{8\mu RT}p_{av}$	ε: porosity; μ: viscosity of the penetrant, η: shape factor; *r:* pore radius; *p*_av_: mean pressure
Knudsen diffusion	2 $D_{k}=\frac{d_{p}}{3}\sqrt{\frac{8RT}{\pi M}}$	*D*_*k*_: diffusion coefficient
	3 $J_{k}=\frac{D_{k}\Delta P}{RTL}$	*J*_*k*_: Knudsen-type flux
	4 $J_{k}=\frac{d_{p}}{3}\frac{\theta }{\tau }\sqrt{\frac{8}{\pi MRT}}\frac{\Delta P}{L}$	
	5 $\alpha_{ij}=\sqrt{\frac{M_{j}}{M_{i}}}$	ε_*ij*_: the selectivity between the species *i* and *j*; *M*_*j*_ and *M*_*i*_: molecular masses of the components *j* and *i*
Selective surface diffusion	6 $W=W_{0}\exp\left[-B{\left(\frac{T}{\beta }\right)}^{2}\log^{2}\left(\frac{P_{0}}{P}\right)\right]$	*W:* the volume of the species adsorbed; *W*_0_: the maximum of adsorbent per adsorbed mass; *B:* the structural parameter; β: the affinity coefficient
Molecular sieving	7 $J_{MS}=\frac{\Delta P}{RTL}D_{i}\left(T\right)\exp\left[-\frac{E_{a,MS}}{RT}\right]$	*J*_*MS*_: molecular sieving flux; *E*_*a,MS*_: molecular sieving activation energy
Solution-diffusion transport	8 P=DS	*P*: permeability coefficient; *D*: diffusion coefficient; *S*: solubility coefficient
	9 $\alpha_{ij}=\frac{P_{i}}{P_{j}}=\frac{D_{i}}{D_{j}}\frac{S_{j}}{S_{i}}$	ε_*ij*_: selectivity between the species *i* and *j*

Precisely, Poiseuille flow is a pressure-driven flow along the length of a channel with a fixed cross-section (*1*). It is a substantial convective flux, which takes place when the pore size is larger than the diameter of the diffusing molecular species. It is therefore unselective, unlike Knudsen diffusion (*2–5*), which exploits the collision of molecules with the pore wall to yield a selectivity proportional to the ratio of the inverse square root of the related molecular weights. Another diffusion mechanism is surface diffusion (*6*) that facilitates the passage through the pore of the most condensable components while excluding the smallest ones. When the molecular sieving mechanism (*7*) takes place, the passage of larger molecules is stopped at the entrance of the pore, while smaller-sized molecules diffuse through it. Lastly, the solution-diffusion mechanism (*8*) takes place in dense membranes, so that molecular diffusivity of smaller species is facilitated through frozen free gaps (*in glassy polymeric membranes*), whereas condensability of molecules with specific affinity to the membrane materials promotes solubility-based separation through fluctuant free volume (*in rubbery polymeric membranes*). Both the mechanisms coexist or become predominant depending on the structure and chemistry of the membrane and permeant.

Each single mechanism could take place within the membrane alone or in combination with others.

### How Membranes Mitigate Environmental Conditions

As a matter of fact, membranes have the ability to transfer selectively molecular compounds from one stream to another, making it possible to regulate humidity levels or remove hazardous airborne substances such as volatile organic compounds (VOCs), carbon monoxide (CO), carbon dioxide (CO_2_), and methane (CH_4_) from specific environments. Some precise parameters need to be considered to establish filtering ability and efficiency of the membrane selected. The main parameters that quantify mass transfer are the flux, which is meant as the productivity of the process, and the selectivity, regarded as an efficiency indicator. The first one indicates how much a membrane can filter within a unit of time and area and is generally expressed as

10 $$J=\frac{V}{A\cdot t}$$

where *V* is the volume flowing through the membrane, *A* is the area of the membrane, and *t* is the time.

The second one is an expression of how much a membrane is capable of removing one species rather than another and is described by the following mathematical relation:

11 $$\alpha_{AB}=\frac{y_{A}/y_{B}}{x_{A}/x_{B}}$$

where *A* and *B* are two components of the mixture, *y*_*A*_ and *y*_*B*_ are the concentrations of two components in the permeate, and *x*_*A*_ and *x*_*B*_ are the concentrations of the components in the feed.

Moreover, the membranes can also work as heat exchangers, combining mass and energy transfer.^20^ This implies their capability to transfer heat and reduce the risks associated with heat islands.

The study concerning heat transfer takes into account a set of equations, which measure various contributions, including a) convective heat transfer from the feed (*heated upstream side*) across the boundary layer to the membrane surface; b) heat transfer across the membrane by conduction and heat accompanying vapor flow through the membrane pores; c) convective heat transfer from the membrane surface at the permeate side across the boundary layer to the bulk of the permeate (*cooled downstream side*). Table 2 summarizes the mathematical eqs 12–14 describing each contribution to the heat transfer.^21^

**Table 2:** Mathematical Equations Describing Heat Transfer through Membranes

Heat contribution	Mathematical equation	Notes
Convective heat transfer from the feed	12 $Q=h_{f}\left(T_{f}-T_{1}\right)$	*h*_*f*_: heat transfer coefficient in feed boundary layer; *T*_*f*_: temperature of bulk feed stream; *T*_1_: temperature at feed membrane surface
Heat transfer across the membrane	13 $Q=N\Delta H_{v}+\frac{k_{m}}{l_{m}}\left(T_{1}-T_{2}\right)$	*N:* permeation flux; Δ*H*_*v*_: heat of vaporization; *k*_*m*_: thermal conductivity of the membrane; *T*_2_ temperature at permeate membrane surface
Convective heat transfer to permeate	14 $Q=h_{p}\left(T_{2}-T_{p}\right)$	*h*_*p*_: heat transfer coefficient in permeate boundary layer; *T*_*p*_: temperature of bulk permeate stream

For eqs 12–14, the values *T*_1_ and *T*_2_ can be calculated at the steady state as follows:

15 $$T_{1}=\frac{h_{m}\left(T_{p}+\left(h_{f}/h_{p}\right)T_{f}\right)+h_{f}T_{f}-N\Delta H_{v}}{h_{m}+h_{f}\left(1+h_{m}/h_{p}\right)}$$

16 $$T_{2}=\frac{h_{m}\left(T_{f}+\left(h_{p}/h_{f}\right)T_{p}\right)+h_{p}T_{p}-N\Delta H_{v}}{h_{m}+h_{f}\left(1+h_{m}/h_{f}\right)}$$

The previous equations consider conduction and convection contributions to heat. However, there is a third contribution to thermal energy flows, which is radiation. In this last case, an important parameter to consider is the emissivity (ε) of the material forming the protective membrane, which is expressed as the ability of a surface to emit heat by radiation. The rate of heat transfer by emitted radiation is calculated according to the Stefan–Boltzmann law of radiation:

17 $$\frac{Q}{t}=\sigma \varepsilon AT^{4}$$

where σ = 5.67 × 10^–8^ J/s·m^2^·K^4^ is the Stefan–Boltzmann constant, *A* is the surface area of the object, and *T* is its absolute temperature in kelvin. For an ideal blackbody, ε is equal to 1, whereas a perfect reflector has ε = 0. Real materials fall between these two values. This implies in a more general sense that all objects absorb and emit radiation. The net rate of heat transfer by radiation is related to the temperature of the protective membrane or monitored object and its surroundings and can be expressed as

18 $$\frac{Q_{net}}{t}=\sigma \varepsilon^{\left(T_{2}^{4}-T_{1}^{4}\right)}$$

where *T*_1_ is the temperature of the object and *T*_2_ is the uniform temperature of the environment. When *T*_2_ > *T*_1_, the quantity is positive; that is, the net heat transfer is from the hot to the cold body.

### Functional Membranes for Active Transport

Over the years, the membrane concept has evolved into new standards of high-definition structure and addressed chemical functionality.^22^ This has allowed us to move from passive to active interfaces, incorporating the concept of assisted transport and responsive functions that are essential for restoration and protection of microenvironments. Membranes have the prerogative to remove and regulate continuously the concentration of pollutants dispersed in the surrounding atmosphere under a gradient of chemical potential, which is generated by a difference of concentration, temperature, pressure, or electrical potential. When particular classes of materials are gathered to form dynamic interfaces, the transport becomes active due to the establishment of amplified and/or stimulated interfacial interactions. Figure 3 summarizes two examples of directed transport associated with particular classes of materials: nanofillers enabling host–guest interactions that accelerate and make the molecular transport more selective (a–b) and responsive systems enabling actuated mechanisms as a response to changes in surroundings (c–d).^22,23^ These kinds of mechanisms could also be combined, leading to intelligent and fast actuation for the reestablishment of targeted ambiences.

**Figure 3:**
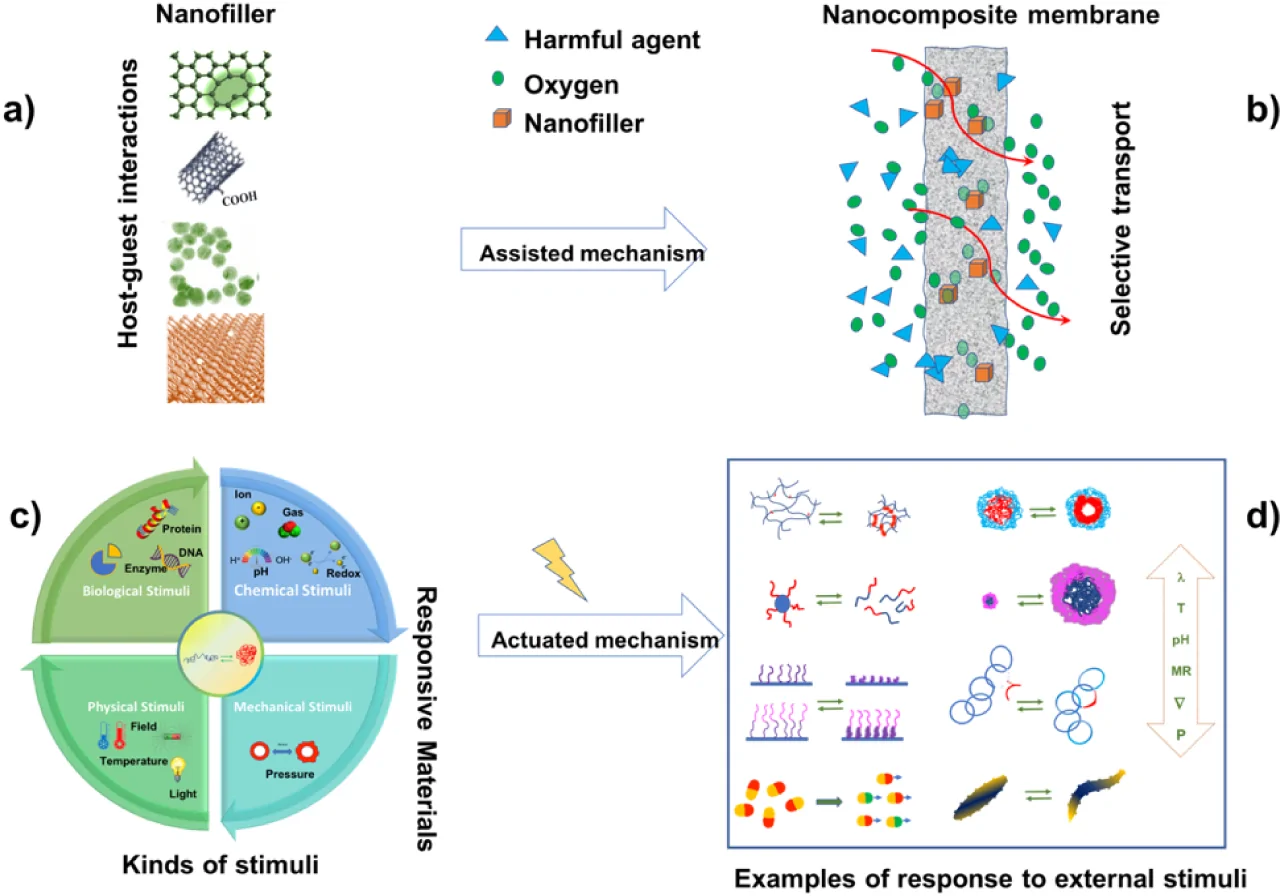
Schematic representation of classes of defective materials for assisted transport (a–b); classes of stimuli associated with responsive materials for actuated mechanisms (c–d).

Regarding assisted transport, the literature refers to a large number of papers focused on the confinement of nano-objects inside polymer networks.^24–28^ Nanofillers—i.e., biomaterials, nanoparticles, functional carbon nanotubes, and defective 2D materials—have been used to amplify mass, energy, and charge transport through host–guest, sorption–desorption, doping, and hopping mechanisms. This approach has allowed the introduction of sites where a quota of permeant can be temporarily allocated, making the transport accelerated.^29^ Hydrophilic and hydrophobic forces, along with vacancies and defects, become therefore powerful tools to address sorption–desorption events while enhancing productivity-selectivity trade-offs.^30–32^

This implies the possibility to design nanocomposite membranes as dynamic interfaces for addressing the quality of each ambience through the manipulated exchange of matter, energy, and charge.

But what benefits can all this bring to the conservative strategy of cultural heritage? One of the most critical factors in preserving art objects and making surrounding environments healthy is the control of humidity levels and temperature, as well as the removal of harmful pollutants. Breathable, adsorptive, and catalytic membranes can be a practical route for regulation of moisture, reduction of critical gases in the atmosphere, mitigation of heat islands, and resistance to physicochemical degradation (Figure 4a–b). The choice to confine nanofillers inside matrixes is expected to amplify the prerogative of membranes to work as efficient and selective air filters and to protect targeted environments proactively. Hereafter, some examples of featured nanofillers are identified as material types for making preventive conservation within scenarios inspired by membrane technology (Figure 4a,b). Gontarek et al.^33^ discussed the water vapor transport coefficient in relation to graphene loaded onto microporous PVDF membranes, confirming that the breathability of membranes can be enhanced by doping mechanisms. Nanofillers such as Bi_2_Se_3_ have been engineered for adsorption of ambient gases, i.e., CO, NO, and H_2_O, yielding changes in the electronic structure of chalcogenides and proving to be interesting for the development of monitoring devices.^34^ Zeolites have been proven to have higher selectivity and activity in catalytic reduction of NO_*x*_,^35,36^ while doping action from donor species has been envisioned in functional carbon nanotubes so that electrical charge and water vapor can be simultaneously transferred, leading to monitored and regulated levels of humidity.^37^ Also, superior photocatalytic activity has been related to defective anatase TiO_2_ nanocrystals for deterioration of organics under visible light irradiation.^38^ In a broader sense, added functionality or defects in nanofillers generate new active sites where adsorption, diffusion, and reactions take place, so that engineered materials can be designed for synchronized monitoring, cleaning, and conservation of works of art.

**Figure 4:**
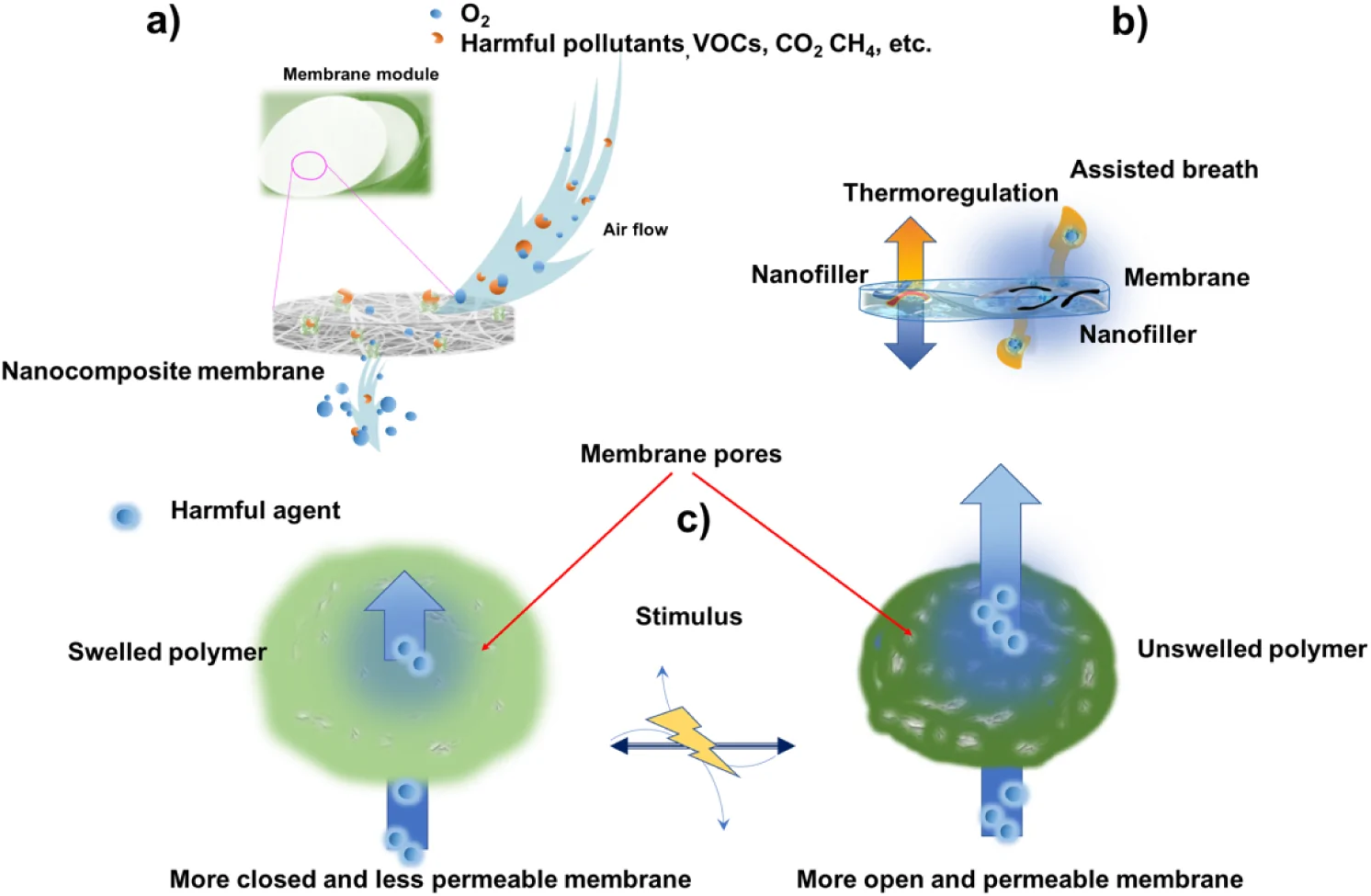
Examples of membrane use in purification and regulation microenvironments: a) nanocomposite membrane for air purification from airborne pollutants; b) membrane for regulation of moisture levels and heat flow; c) responsive membranes enabling actuation mechanisms triggered externally to decrease or increase the diffusion of harmful agents dispersed in air.

Intelligent membrane-based devices are further fast-acting solutions and can be developed through the interplay of materials with associated adaptive functions. This category of materials consists of responsive compounds with the ability to switch and adjust themselves reversibly under external trigger actions.^39^ Morphology, charge, and chemistry of materials can be manipulated by using switchers such as light, temperature, pressure, pH, fields, and molecular recognition (Figure 3d). This kind of membrane has the ability to work on demand and subsequently regulate the purification process when necessary (Figure 4c).

Widening, usability, and flexibility of these classes of materials depend on the complexity of their structure and chemistry. Single or combined triggers can be used in order to produce hydrophilic–hydrophobic transition, volume shrinkage-expansion, phase transition, surface charge variation, color change, etc.

But the question is how these materials can impact artwork conservation. The use of responsive membranes is expected to become a powerful tool for revealing local environmental variations and working as real-time monitoring sensors. The responsive mechanism involves normally a reaction such as deprotonation/protonation, hydrogen bonding, electrostatic and van der Waals interactions, cross-linking, isomerization, bond formation or cleavage, and charge transfer.^40^ The resulting effect is tendentially a change in shape, conformation, assembly and disassembly, electronic or optical properties, *etc.* However, if these kinds of functions are placed in microarray-like membranes, more complex sense-to-act events take place, so that hazardous and unsafe physical, chemical, and biological events could be inspected, contrasted, and neutralized synchronically and as required.^23^ This is expected to become a key element in the design of next-generation smart devices for assisting the restoration and conservation of artistic settings and generating buffer zones and controlled surrounding environments. Hereafter, a scenario of some practical applications of membranes in cultural heritage conservation is analyzed.

### Structural Membranes for Outdoor Shielding

Membranes find an interesting outdoor application in refurbishment, protection, and creation of sheltered spaces, providing useful solutions for comfort and energy-saving without sacrificing the aesthetics of historic buildings. Lightweight, flexible, reusable, and removable structural membranes, based on textile materials—polyvinyl chloride (PVC), polydifluoroethylene (PVDF), or polytetrafluoroethylene (PTFE)—are frequently used for shielding architectures in a noninvasive way. They can have different geometries, modular shapes, and a low visual impact but with a great ability to withstand adverse weather conditions. Additionally, membranes can be removed so that potential damage to the original structure is minimized. Figure 5 shows an example of membranes used as a transparent roof to protect the ruin of Corbera d’Ebre Church, located in Spain, without affecting, however, the historical value of the monument.^41^ This is an example of how transparent membranes offer protection against external agents while making the monuments still usable for visitors.

**Figure 5:**
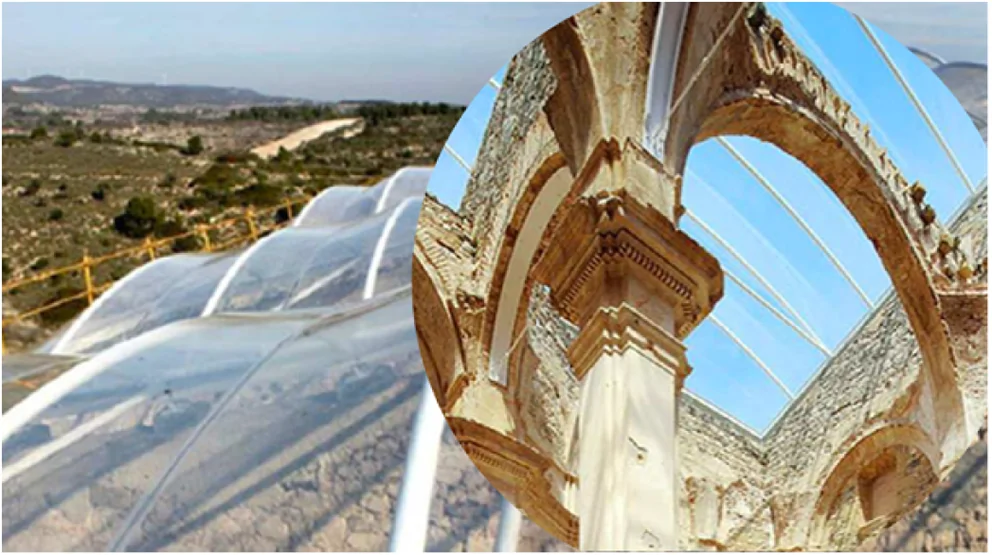
Valley cables stretched membranes that provide a transparent roof to maintain the character of the *Corbera d’Ebre Church* (Spain) ruin. Adapted from ref 41. Copyright (2016), with permission from Elsevier.

Today, there is a lot of attention toward advanced materials for the fabrication of membranes to be used as sustainable tools for preventive protection of archeological sites, historical buildings, fountains, statues, and precious portals, *etc*. They can provide different levels of protection against light, rain, wind, snow, cold, or warmth in order to reduce corrosion, condensation, humidity, and heat variability, as well as fluctuations of frost and thaw during the winter season.^41–43^ Depending on the final use, i.e., coverage, internal or attached space, membrane structures can be classified as open, enclosed, and convertible structures and are aimed at providing comfort to visitors and monuments through a modular exchange of light and air.^44,45^ Computational fluid dynamics have been used to simulate the effects of membrane design and the surrounding environment on air distribution, relative humidity, air and surface temperature, and acoustics as well.^46,47^ It has been demonstrated that membrane design can alter the airflow, thereby producing changes in air quality and thermal comfort. A predictive study based on dynamic simulations has revealed the effects of membrane textiles installed on the roofs of a historical building in L’Aquila (Italy).^45^ Reductions of CO_2_ emissions (i.e., 8.8% in January), energy consumption (i.e., 5.5% in August), and natural gas consumption (i.e., 29.2% in March) have been confirmed for ambiences protected with textile roofs. A similar approach has been proposed to protect Pompeii’s frescoes and mosaics from solar incidence, yielding comfortable ambience for visitors with thresholds of temperature in the range of 18–20 °C and relative humidity of 40–60%.^48^ Multilayer membranes consisting of polyester (PES)/polyvinyl chloride (PVC) or glass/PTFE have been also examined as better thermal textile protection due to less heat transfer and reduced energy loss, but even more as solutions to get relatively high transparency (>50%) in combination with material strength of up to 60 kN/m. The major benefit derives from the possibility to get a different geometry—due to the flexibility of materials—that combined with related transparency allows receiving natural light from outside during the day, whereas radiating internal light through the semitransparent materials during the night so that the bright crystal monuments are perceived. This kind of solution has been also proposed for the protection of modern architectures such as the Fuji Museum and Hazza Bin Zayed Stadium in Al Ain (Figure 6), without affecting the original design and meeting the request to be removed at any time.^49^

**Figure 6:**
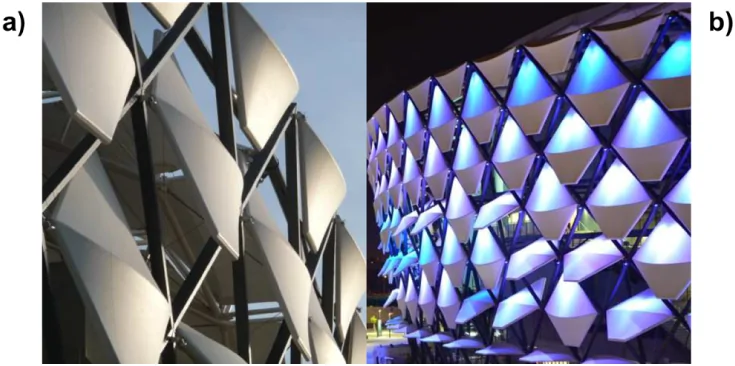
Hazza Bin Zayed Stadium in Al Ain: (a) Glass/PTFE panel for external shading; (b) illuminated membrane panels. Reprinted from ref 49. Copyright (2016), with permission from Elsevier.

Diffusing natural light toward the building space reduces energy consumption. However, it is important to use materials with high reflectivity on the outside surface and low radiation coating on the inside surface in order to reduce radiation heat exchange between the membrane surface and the indoor environment. For outdoor applications, a membrane-assisted radiant cooling system has been proposed as an alternative, which directly treats the radiant loads while minimizing the convective heat loss to the ambient.^50^ Specifically, a sky-window selective membrane has been demonstrated to allow only transmission of infrared in the 8–15 μm wavelength range, with energy saving of up to 44% and 47% and a reduction of the convective heat transfer coefficient of the cooling panel in the range of 2.2 W/m^2^ K to 2.6 W/m^2^ K. In this respect, structural membranes do not exhibit always thermal resistance; rather, they can promote heat transfer and reduce significantly the temperature gradient. An interesting example is that of lightweight textile membrane canopies, which have been proposed to create outdoor thermal comfort in towns suffering from very hot summer climate such as the city of Semnan in Iran.^51^ In this context, simulations based on different values of relative humidity, air temperature, and wind speed have demonstrated that a membrane canopy of 25 m^2^ and a height of 3 m produces a reduction of temperature of up to 7.8 °C, limiting the environmental impact to 40%. It is crucial to underline how the design of membranes from different classes of materials is evolving, but predictive heat transfer mechanisms are still necessary to address their use in outdoor contexts. Recently, 2D materials-based PVDF membranes have been proposed as interesting interfaces enabling water vapor transfer (WTR up to 1170 g^–2^ day^–1^@55 °C) to be combined with a different degree of heat diffusion.^52^ In particular, it has been demonstrated that few-layer graphene entrapped in a PVDF matrix promotes heat flow of 10^6^ W m^–2^, whereas a reduction of heat diffusion can be obtained with few-layer Bi_2_Te_3_ (10^4^ W m^–2^). Accordingly, the use of nanocomposites is expected to pave the way for real solutions to keep comfortable ambience around monuments and artifacts, especially if regarded as vulnerable. Indeed, glass and textile membrane-based protections of artistic assets such as masonry works, i.e., mosaics, frescoes, and wall painting, are envisaged to be a valid alternative to the risky removal from their original place. These kinds of artifacts are heavily exposed to harsh weather and often suffer irreversible effects from salt crystallization and biodeterioration due to uncontrolled microclimatic conditions. For this reason, structural membranes could be used to shelter partially them and generate buffer zones and monitorable surrounding environments, providing waterproofness and breathability to the ambience. A study carried out on historical buildings with medieval wall paintings on the Croatian Adriatic Coast has emphasized the urgency of using external protection, rather than the risky removal of paintings from their original place, for preserving the frescoes from the growth of fungi and molds as well as salt contamination (MgCl_2_, MgSO_4_, CaCO_3_, NO_3_^–^), which are triggered by high humidity, temperature, and light.^53^

## Membranes for Indoor Environment Mitigation

One of the major concerns for the conservation of artworks within an indoor setting is the necessity to establish ideal climate conditions to counteract deterioration due to negative effects ascribed to climate change, overtourism, or unsuitable management of exhibition rooms, or more generally the ambience. Temperature and humidity levels, suitable light intensity, and safe air quality need to be discerned to preserve the surroundings of each single object. All of these decay factors work often together, leading to complex interactions that generate degradation processes around the artifacts at different length scales.

### Targeted Ambiences and Critical Issues

The management of climatic conditions related to an indoor ambience could appear easier than outdoor ones. Really, concomitant factors can make it somewhat difficult and unpredictable over time. Fluctuations of humidity, temperature, heat, and CO_2_ levels together with other physical, chemical, and biological contaminations are among the major causes of imbalance in a natural confining environment, which surrounds artifacts. Depending on the volumetric space that characterizes the surroundings and the type of material to be conserved, calibrated and addressed actions are hence needed. Concerning humidification, dehumidification, heating, cooling, particulate filtration, noxious gas filtration, and microbiological control, numerous studies have been proposed over the last decades.^54–60^ In parallel, advanced sensors and microprocessors are increasingly proposed for real-time monitoring.^61–67^ A recent study has been carried out for identifying a magnitude index needed to assess the microclimatic risk within Villa Barbaro, a monument built in Maser between 1554 and 1560 by the architect Andrea Palladio (UNESCO World Heritage Site, 1994).^68^ This building houses frescoes, stone for frames and capitals, as well as also precious wooden doors and windows that require selected and diversified microclimate conditions. In this study, sensors integrated with IoT technology have allowed indoor building monitoring over six months, while a virtual building model has been realized to predict various scenarios, taking into account the role of seasonal weather and visitors. In another widespread study, monitoring of various environmental scenarios, including the Fondazione Scienza e Tecnica, Florence (FST); Peggy Guggenheim Collection, Venice (GGH); Chieti State Archive, Abruzzo (MB) in Italy; Centre Georges Pompidou (PPD) and Quai Branly Museum (QB) in Paris, France; National Museum of Slovenia, Ljubljana (NMS), and Hungarian National Museum, Budapest (NMH), has been proposed.^69^ Effects of seasonal changes on the surroundings of galleries/display rooms, storage rooms, display cases, and storage enclosures such as boxes, crates, and cupboards have been investigated, and a lot of data have been collected, yielding insights into the role of territory—countryside and industrial areas—, building size and architecture, seasonal weather, and pollutant sites.

Despite the fact that monitoring provides important information about decay factors and related fluctuations, it needs, however, to be associated with devices that can reestablish targeted air quality for each kind of environment and its surroundings. Monitoring reveals but does not prevent peaks of atmospheric variables nor does it allow real-time diversification of environmental conditions per single object. The practice to keep the indoor pressure of buildings and museums higher than that of the outdoors is not useful. The artificial atmosphere is not in equilibrium with walls, floors, and ceilings, resulting in continuous additions and subtractions of heat and moisture to balance surfaces with the surrounding air. Constant conditions close to natural ones, especially during seasonal cycles, should be ideal for preserving objects as well as possible. Modern climatic machines could apparently operate well, but adverse events can take place, producing stress and damage to collections and objects over time. Clouds of moist/dry, cool/warm air can be generated and moved in an uncontrolled mode in proximity to the works of art, leading to local fluctuations and perturbations in the atmosphere. Additionally, a huge amount of energy is spent because of significant heat loss.

Another critical issue is related to the establishment of uniform and appropriate guidelines. Rather than materials conservation, comfortable ambience standards are tendentially assimilated to human well-being according to the following regulations: ISO 7730, 2005; EN 15251, 2007; ASHRAE STD 55, 2017. Indeed, microclimatic conditions for objects are not often compatible with visitors’ comfort.^70,71^ Hereafter, some guidelines are provided to preserve objects from deterioration. Table 3 displays the different moisture levels required by various classes of materials and objects, while Table 4 summarizes more appropriate relative humidity and temperature per material type.^72^ Specifically, Table 3 emphasizes how not all objects can be stored in the same environment due to their different chemical nature and vulnerability to external humidity.

**Table 3:** Classes of Materials and Related Stability to Relative Humidity and Temperature

Requested moisture levels	Materials
Extremely stable	Lacquared furniture, oil painting, wooden musical instrument, wood carvings, paper and parchment, Japanese screen, plaster
Moderately stable	Clothes, costumes, wooden polychrome objects, ivory, horn, leather and skin garments, Chinese lacquered object
Insensitive	Stone, marble, ceramics, silver, gold alloys
Dry	Iron, steel, bronze, textiles with metallic element, mummified exhibits, glass

**Table 4:** Recommended Temperature (*T*) and Relative Humidity (RH) for Some Artifact Types Storage^72^

Object	*T* range (°C)	RH range (%)
Painting	20	45–55
Paper	20	30–60
Glass	13–18	30–40
Photographs	13–16	30–50
Film (Acetate cellulose)	–10 to 2	50–30
Film (Polyester)	13–21	30–50
Film (Cellulose nitrate)	–18 to 2	20–30
Optical media	20	30–50

In Table 4, more precise and diversified temperature and humidity ranges are reported for different classes of objects. Specific microenvironments are recommended for preserving them over time.

Table 5 shows in a general way the maximum allowable values of pollutant concentrations in indoor ambiences, although these should be considered as general guidelines since they have been elaborated without considering synergistic effects of numerous variables, specificities of single objects, and diversified storage locations.^69,73^

**Table 5:** Guidelines for Maximum Allowable Concentration of Airborne Pollutants in Museums (μg m^–3^)^69,73^ ^b^

Airborne pollutant	General collections	Sensitive collections	Long-term storage	Refs
Acetaldehyde	-	2–37	-	^68^
Acetic acid	100–700	12.5	2500/250 for sensitive	^68^
Formaldehyde	12.5–25	0.1–6	375	^68^
NO_2_	4–20	0.1–5	20	^68^
SO_2_	1–5	0.1–1	3	^68^
Hydrogen sulfide^a^	0.14	0.014	0.1 (*10 years*)^b^	^72^
O_3_ ^a^	1.0	0.1	1 (*10 years*)	^72^
PM_2.5_ ^a^	1	0.1	1 (*10 years*)	^72^
TVOCs^a^	-	1.8	-	^72^

a Adopted by Getty Conservation Institute (GCI).

b Adopted by Canadian Conservation Institute (CCI).

It is somewhat unquestionable that to get uniform and fine indicators for everything is quite difficult; but once the ideal ambience for each class of materials has been identified, the best practice should be to keep constant all surrounding conditions through real-time counteraction to undesired events. Using artificial intelligence (AI) tools could well help to identify preliminary optimal environmental conditions for each class of objects; however, engineered materials remain a needed practical route to reach and keep constant surrounding environments.

### Gas/Vapor Removal

Materials science provides numerous engineered solutions to design devices enabling microclimate control, especially in textiles, medicine, and greenhouses;^74–76^ however, there are a few examples of systems tested to perceive changes in surroundings and provide buffer climatic zones near cultural heritage assets.

Figure 7 shows the concentration of pollutants such as acetic acid (AcOH), formic acid (HCOOH), formaldehyde (FDH), acetaldehyde (ADH), and NO_2_ in different European Museums: **a)** Abruzzo (MB), Budapest (NMH), Ljubljana (NMS), Quai Branly (QB); **b)** Florence (FST), Venice (GGH), Centre Georges Pompidou (PPD), revealing how seasonal effects, locations, and the kind and size of environment displays yield different and complex contexts that require targeted mitigation actions.^69^ These data are the result of long-term monitoring of different ambiences that are affected by the effects of the territory, flows of tourists, climate, and latitudes. The complexity and multifaceted nature of the data confirm how much each specific environment requires particular and flexible protective measures and how similar objects need not be protected in the same way but rather in relation to the specific environment in which they are immersed. On this basis, it is necessary to identify best practices based on the use of categories of technologies and materials capable of regulating certain environments according to specific needs.

**Figure 7:**
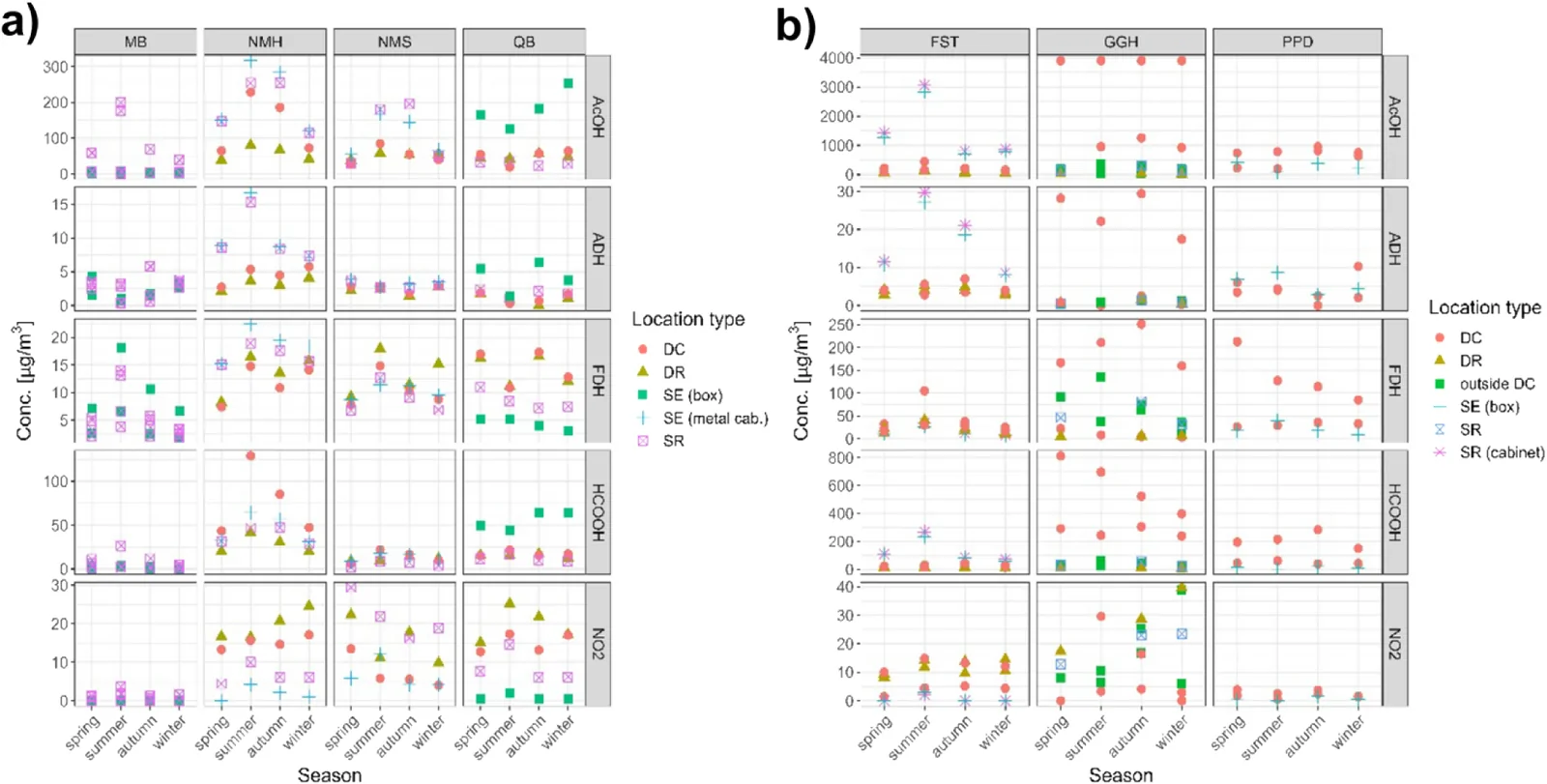
Fluctuation of pollutant concentration collected across seasons at different museums located in European Countries location: a) MB, NMH, NMS, QB; b) FST, GGH, PPD. Reprinted from ref 69. Copyright (2024), Springer (Creative Commons CC BY license).

Clay minerals, including kaolinite, montmorillonite, and illite, have been, for example, proposed as compounds to promote passive sorption/desorption of moisture, maintaining RH within a range of 35 to 65% over 12 h.^77^ However, they are not long-term stable compounds and exhibit only 27% of the total sorption capability for a room with a volumetric space of 60 m^3^. In a similar way, passive mitigation actions based on various classes of materials, i.e., VOC absorbers, graphene coatings, *etc.*, have been proposed to improve the air quality of various museum environments.^69^ These materials require, however, replacement or reactivation once exhausted. Despite the fact that fouling events must be carefully checked for greater efficiency, membranes are envisaged as a valid alternative to traditional absorbers because they associate sorption and transfer in a one-pot stage, resulting in a continuous and selective purification process.^22,37^ So, membranes can regulate the levels of gases and vapors dispersed in air continuously and to varying extents.^78,79^ As an example, CO_2_ can be removed by using nanocomposite hybrid membranes, i.e., polyimide, Pebax, polysulfone in combination with alumina, silica, zeolite nanofillers, graphene, graphene oxide, and carbon nanotubes, through selective sorption and diffusion mechanisms. Table 6 summarizes the performance of some membranes in CO_2_/N_2_ separation, providing indication about the possibility of subtracting excess CO_2_ from air when facing environments enriched in it.

**Table 6:** Nanocomposite Membranes and Related Affinity to CO_2_

Membrane Polymer/nanofiller	Nanofiller loading (wt %)	CO_2_ permeability (Barrer)	CO_2_/N_2_ selectivity	Reference
Polysulfone graphene oxide/ZIF-301	5/6	25	64	^80^
Polyimide SO_3_H-MCM-41	30	9	32	^81^
Polyimide graphene oxide/CNT	5/5	36.5	76	^82^
Pebax Zeolite 4A	20	113.7	39.5	^83^
Pebax ZnO	8	152.3	62.2	^84^
Pebax graphene oxide	10	260	60	^82^
Pebax triethylcitrate	70	405	35	^85^

The data listed in Table 6 yield clear indications about what can be done regarding microenvironment mitigation. The confinement of nanofillers in polymer matrixes allows for the selective removal and permeation of a critical gas, such as CO_2_, for continuous purification processes.

Membranes have, however, a great ability to remove selectively and proficiently other kinds of gases and VOCs from air. The literature refers to many contributions on the use of membrane technology to remove air pollutants such as SO_*x*_, HCl, NO_*x*_, CO, Freon, Halon, toluene, xylene, trichloroethylene, trichloroethane, and ethylene glycol monoethyl ether.^86^

Anyway, the membrane’s ability to work as a dynamic interface for on-demand microclimate and microenvironment regulation can be aimed if smart materials are used to design them.^21^ As an example, CO_2_ has been also proposed as a stimulus to modulate reversibly the surface and other properties of materials.^87^ It has been demonstrated that gaps in membranes can be opened or closed through the reaction of protonation/deprotonation involving tertiary amines (−NR_1_R_2_) and CO_2._ But there is not yet any applicative evidence of this kind of material in the cultural heritage context.

### Heat and Humidity Regulation

Block polyurethanes (PU), whose hard segments are dispersed in soft regions, have been proposed to realize fibrous membranes for providing bidirectional thermoregulation against heat changes in the surroundings.^88^ These systems, which are regarded as energy storage materials (4.1 kJ m^–2^), counteract local overheating or overcooling and subsequently they activate when necessary. Change phase materials (PCMs) have been also embedded in silicone films for thermoregulation of microenvironments. Specifically, their capability to loss and gain energy has been investigated in proximity to a wooden painting stored in the S. Croce Museum in Florence, Italy.^89^ Interestingly, it has been envisaged the opportunity to locate PCM materials at a suitable distance from the object in order to achieve a good balance between energy storage effectiveness and reduced thermal gradient. This is because large gradients could affect the objects over time, especially if they are sensitive to thermal shocks. Low thermal conductive materials such Bi_2_Te_3_ have been also proposed to reduce heat loss through the membrane interface, preserving a great breathing capability.^52^ Thermal insulation needs indeed to be combined with permeability and breathability properties in order to prevent condensation. The latter is regarded as the main cause for biodeterioration, corrosion, crumbling, cracks, tears, and loss of materials. Graphene oxide (GO)/poly­(vinyl alcohol) (PVA) composite films have been proposed to regulate moisture (RH up to 87%) under strong temperature fluctuations (Δ*T* = ±24.1 °C) inside small and medium-sized museums, libraries, or archives, proving effective also for acetaldehyde adsorption up to 45%.^90^

Carbon nanotube-based membranes have been proposed to perceive changes in relative humidity and regulate its levels inside microenvironments.^37^ The membranes have been fabricated by combining a pH-responsive hydrogel such as PAA with functional single-walled carbon nanotubes (SWCNTs).^91,92^ According to the layer-by-layer method, pristine and functional CNTs have been deposited using pH as a trigger in order to get filaments in an exfoliated state, thereby forming electrical networks with the widest possible number of contact points.^91^ The number of contacts among the tubular filaments has allowed significant charge transfer throughout the surface, allowing the membranes to work as detectors of humidity fluctuations. Experiments carried out inside a climatic chamber have simulated typical indoor building (*T* = 22 °C, RH= 40–50%), subtropical climate (*T* = 22 °C, RH = 70–80%), and saturated ambient (*T* = 22 °C, RH > 90%) conditions. Fluctuations of relative humidity within a range of 5 to 10% have caused a variation of electrical conductivity of 7% in less than 300 s, confirming high interfacial sensitivity. In parallel, it has been demonstrated that the membranes have the capability to transfer concurrently large amounts of water vapor −1400 g^–2^ day^–1^ (*T =* 40 °C; RH = 50%) to 1000 g^–2^ day^–1^ (*T* = 40 °C; RH = 80%)—under gradients of partial pressure.^37^ These studies confirm the possibility to merge in the same membranes features of moisture sensors and regulators, although their potential to generate buffer zones in proximity to works of art has been not yet inspected under real conditions.

Similarly, a monolithic membrane based on PVDF, Pluronic F127, and TiO_2_ nanoparticles (PVDF@F127-TiO_2_) has been proposed as a humidity actuator with an ultrafast bidirectional response (10 s), bending deformation of 239.1°, and tensile strength of up to 27.7 Pa, and high thermal radiation with a mid-infrared emissivity of 94.3%.^93^ The possibility to convert chemical energy into kinetic energy has been exploited to open gaps through which airflow and regular breathing can occur by thermoregulation mechanisms. Accordingly, membranes based on polyamidoamine (PAMAM) bridge-enhanced carboxylated holey GO (hGC/PAMAM) have been designed to promote breathability through swelling mechanisms. Asymmetric expansion along the horizontal and vertical directions has been proven to open gaps for the rapid transfer of water vapor under gradients of humidity.^94^ Also, elastomeric membranes containing IL [C_12_C_1_im]Cl have been suggested for reversible thermoregulation of microenvironments, achieving up to 40 g^–2^ day^–1^ m at 40 °C and 7.358 kPa.^95^ The IL has been confined and thermally driven to generate preferential water vapor diffusion pathways along the nanofiller.

In a stricter applicative sense, these membranes exhibit features suitable for regulating moisture levels in confined spaces and could equip dehumidification systems for a sustainable museum ambience.

However, some engineering aspects must be considered. As is known, membrane technology is recognized for giving versatile and energy-saving solutions due to its scalability, compactness, and sustainability. In this logic, Liu et al.^96^ have proposed modular system solutions for precise humidity regulation in museum cabinets, suggesting an approach based on integrated multiparameter modeling using a response surface methodology (RSM) and an optimized genetic algorithm (GA) (Figure 8). Figure 8 shows a compact and scalable membrane system designed to investigate the role of materials, environmental conditions, and operational parameters in regulating a microenvironment around a cultural relic. It is quite interesting to observe how hollow fiber membranes have been assembled into a module and put in contact with humid air, allowing the airflow to pass from the shell to the lumen under a difference of pressure generated by a vacuum pump. In this study, exclusive needs such as strict spatial constraints, ultralow airflow noise, and stringent humidity stability have been considered in order to solve some critical issues related to materials’ hygrothermal behaviors, net-zero carbon compromise between conservation efficacy and energy savings, and adaptability of the system to specific material-dependent needs. Fluid dynamics simulations of airflow and humidity distribution, along with membrane mass transfer kinetics have been carried out in order to study moisture exchange between artifact environment interfaces and membranes, while humidity flow inside the cultural relic cabinet has been analyzed. An RSM empirical model has been used to investigate the impact of operational factors, including inlet airflow, relic relative humidity content, filling rate, and the structure of the relic cabinet, as well as membrane area. The system configuration parameters have been hence optimized with a genetic algorithm (GA) multiobjective optimization, including average humidity inside the relic cabinet, dehumidification efficiency, and dehumidification capacity per unit membrane area.

**Figure 8:**
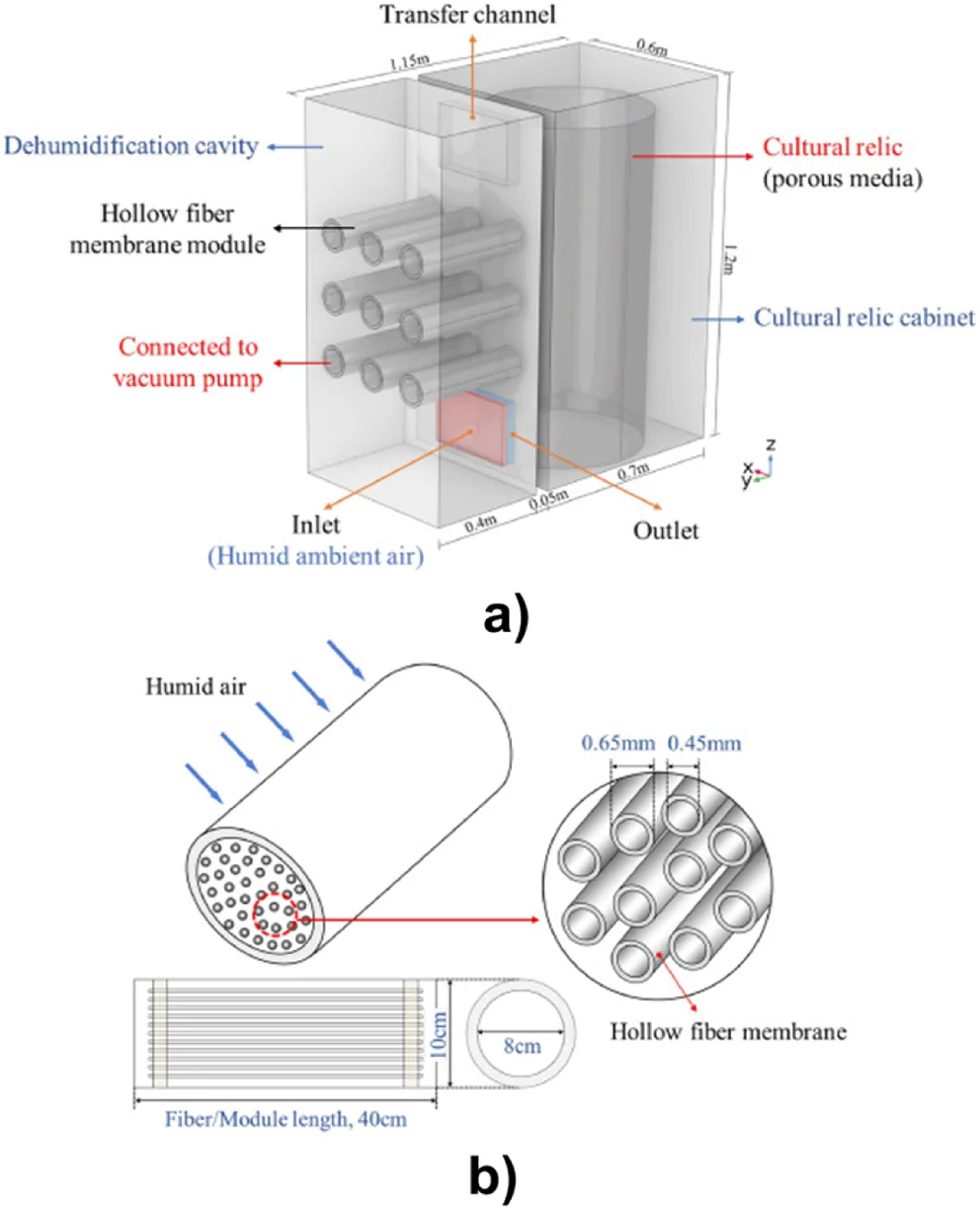
Modular dehumidification system for heritage storage cabinets (a). Dehumidification membrane module (b). Reprinted from ref 96. Copyright (2024), with permission from Elsevier.

As a result, a gentle and effective humidity control has been achieved, moving progressively from convective-diffusive to diffusion-dominated transport so that targeted humidity conservation ranges have been met, while inlet humidity, feed rate, and permeability coefficient have been envisioned to be key parameters in the dehumidification process, although there is an interdependence between operational factors and material properties. This study can be regarded as a real application of membrane technology for regulating the humidity levels inside a cabinet and highlights how material science needs to be integrated with process engineering through the interplay of functions and operational parameters, which requires evaluation of object materials, the space where the object is allocated, and local environmental settings.

### Powder Filtration by Membranes

Membranes can also play a key role in air decontamination and sanitization. They can capture particulate matter from the air and also prevent the diffusion of bacteria and virus in the air. As is known, particulate matter (PM) can be classified into PM_0.3_, PM_0.5_, PM_1.0_, PM_2.5_, and PM_5.0_ depending on their size within the range of micrometers. PM represents a serious risk to human health, but it is also regarded as a hazardous pollutant due to its chemical aggression and the soiling of works of art.^97^ A composite membrane of poly­(vinyl alcohol) and Eugenol (Eo), which is a hydrophobic aromatic compound found in natural essential oils, has been proposed to filter PM_1.0_ and PM_2.5_, with efficiencies of 99.74 and 99.77% and quality factors (QFs) of 0.0351 and 0.0358 Pa^–1^, respectively. An efficiency of 99% has been assessed under high humidity levels, making it an interesting material for green air filters to place in ventilation devices.^98^ MOF Nanosheet-Functionalized Poly­(lactic acid) Meta-membranes have been also realized for long-term air purification and intelligent monitoring,^99^ achieving a filtration efficiency of 99.2% for PM_2.5_ and 96% for PM_0.3_ with an airflow rate of 85 L/min. In this case, triboelectric performance and a self-charging mechanism have made the membrane a specific sensor of airflow, allowing real-time monitoring of air quality.

### Biological Decontamination by Membranes

Another concern is the biodeterioration of paper, wood, glass, stone, leather, textiles, painting caused by macro-organisms such as bacteria, archaea, fungi, and microalgae, as well as lichens, plant mosses, and insects.^100^ In the past, quaternary ammonium salts, ethylene oxide, alcohols, aldehydes, halogen compounds, peroxides, surfactants, and heavy metals were used to sanitize artifacts. Many of these chemicals are unfortunately hazardous and unsafe for people and can be aggressive toward sensitive works of art. Today, new materials are proposed to counteract biodeterioration, including Cu, Au, Ag, TiO_2_, ZnO, SiO_2_, and CuO NPs, along with graphene and its derivatives, which, in addition to having antifungal and antimicrobial properties, exhibit the great feature to strengthen and consolidate artwork materials. However, NPs and other nanofillers can be easily integrated into membranes and work as biocides through temporary and removable coatings^101^ and when equipping devices for air filtration, so that the spread of airborne pathogens is prevented.^102^

The interfacial and colloidal chemistry provide advanced solutions to remedial conservation; they make it possible to overcome some limitations due to the utilization of traditional materials in matters of compatibility, time-consuming procedures, and hazardous risks. The dispersion of nanoparticles has been explored and successfully applied to conservation of Classical Age and Renaissance artifacts;^103^ however, the chance to preserve the surrounding environment from pollution through the confinement of NPs in hosting matrices for air decontamination is an attractive topic. Nanofibrous filters are generally preferred to make the filtration of smaller particles more efficient when a slight increase in pressure drop is applied. Each single fiber can be functionalized with biocidal agents in order to intercept airborne pathogens and destroy them in situ so that the same filter is preserved from becoming a source of contamination.^104^

While cellulose acetate, polyesters, polyacrylonitrile, polyamide, and polypropylene are the most selected polymers for the fabrication of filters, biocide agents include oxides such as MgO, TiO_2_, and Al_2_O_3_,^105^ and silver NPs.^106,107^ As an example, biohalloysite has been proposed for polypropylene/polyester (PP/PET)-based air filters for air decontamination from *Escherichia coli*, *Candida albicans*, *Aspergillus niger*, and *Staphylococcus aureus*.^108^ For the latter, the efficiency has been 99.86%. Butanetetracarboxylic acid (BTCA) has been cross-linked with poly­(vinyl alcohol) (PVA) nanofibers to decontaminate air from PM_0.3_, PM_0.5_, and PM_3.0_, with an efficiencies of 85.84%, 98.37%, and 99.79%, respectively, from *E. coli* and *S. aureus* with a reduction of 1.9 and 2.5 log units after 2 h of contact, and from coronavirus HCoV-229E with a reduction of 2.0 to 3.0 log units after 1 h of contact and an efficiency of 99%.^109^

More recently, essential oils (EOs), i.e., terpenes, phenylpropanoids, and organic compounds with sulfur and nitrogen moieties, have been envisaged as useful tools against biological contamination of air. Cinnamon essential oil in combination with activated carbon (AC) has been proposed as a biocidal agent inside PU filters.^110^ Similarly, carvacrol (oregano), menthol (mentha), camphor (cinnamomum), linalool (coriandrum), and thymol (thymus) have been investigated as potential bacterial and fungal spore inhibitors, even though the mechanisms have not yet been explained fully.^111^ It is relevant to stress that EOs need to be encapsulated in polymeric matrixes before being incorporated into air filters. This step is required to preserve their chemical, mechanical, and thermal stability. On the other hand, their distribution inside the film inhibits the formation of biofilm and clogging throughout the filter surface, thus preserving long-term efficiency.

### Membranes against Infiltration and Salt Crystallization

Another concern is the destructive process induced by moisture absorbed in porous materials.^112^ Humidity triggers salt crystal formation, causing cracks, breaks, and also corrosion. Membrane science offers valid solutions to counteract humidity infiltration and salt migration. Hydrophobic porous membranes prevent water condensation and concurrently remove the excess moisture by evaporation. Hydrophobicity, as a result of chemistry and morphology manipulation, is combined with reduced resistance to transport so that waterproofness is associated with high breathability.^113^ Membranes with these features are usually proposed for desalination through the implementation of membrane distillation^114^ and membrane crystallization^115^ operations. For these processes, the membrane has the prerogative to generate a liquid–gas interface at the entrance of each pore, preventing liquid water from wetting and allowing the water vapor to diffuse under a gradient of temperature, while salts, which are nonvolatile substances, cannot migrate through the protective layer. Hydrophobic membranes can, however, experience wetting over time due to contamination by low surface tension substances. While efforts to make the membranes superhydrophobic and omniphobic to counteract wetting are importiant, water condensation can still occur, leading to a loss of protective efficiency. Drying processes to reestablish the membrane’s performance can be obtained by applying eternal forces such as hot air or a vacuum oven.^116,117^ These practices cannot be, however, proven effective in contexts similar to those needed for cultural heritage protection. So, a spontaneous drying process has to be induced.^118,119^ Gu et al.^120^ have proposed a composite hydrophobic membrane to stimulate self-dehydration of the pores through a difference of adhesion between hydrophilic and hydrophobic materials to water, without the necessity to use external forces.

They have exploited the concept of the critical wetting depth value (δ) of hydrophobic membrane materials, as well-explained in Figure 9. When the penetration of pure water into membrane pores is less than the critical wetting depth value, water molecules self-assemble into clusters due to repulsion against hydrophobic neighboring surfaces. As a result, they are pulled out spontaneously under the interfacial tension action. This spontaneous dehydration with the advantage of efficiency saving depend however on the hydrophobicity of the material forming the membrane, pore size, and surface roughness. In any case, these kinds of waterproof and breathable membranes are today commercialized as tapes or spray formulations for retrofitting heritage buildings, masonry works, or constructing timber frames and offsite builds.

**Figure 9:**
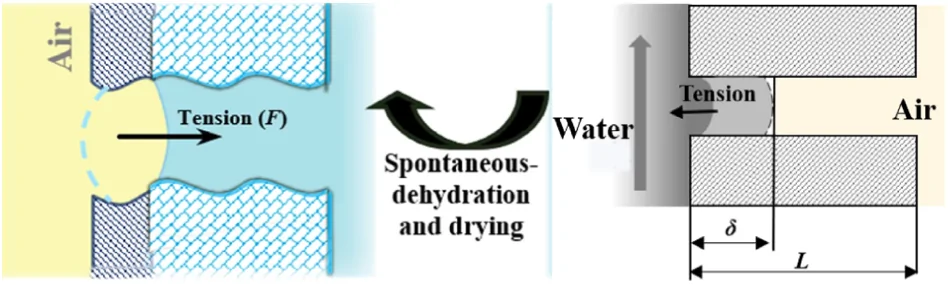
Representation of spontaneous dehydration and drying, and the critical wetting depth. Reprinted in part from ref 120. Copyright (2025) with permission from Elsevier.

## Conclusions and Perspectives

Despite the fact that membrane science and technology-based solutions are still little exploited within cultural heritage assets, they represent a fruitful chance to counteract unbalanced variables and harmful, unsafe agents regarded as responsible for degradation. Membranes can reestablish physicochemical variables and also neutralize or remove chemical, physical, and biological pollutants through different mechanisms; they can restore and keep constant surroundings considered ideal for each type of artifact. Membranes provide outdoor solutions as structural protection for historical buildings and monuments, creating sheltered spaces against weather changes, corrosion, and thermal excursion, while providing comfortable conditions. They can work in indoor ambience as controllers of vapors and gases diffusing through air, regulators of heat and moisture levels, and they can isolate historical assets from dust, biological pollutants, and water infiltration. Membranes have the advantage of being modular, scalable, adaptable, and easy to coat and remove at any time so that the aesthetic, original structural, and integrity of the artifacts are preserved in various contexts. However, their application in real-world scenarios requires careful integration of materials science and process engineering knowledge, together with access to environmental data collections.

Herein, the main advancements and perspectives of membrane applications within the context of preventive conservation for cultural heritage are examined. Today, a lot of studies propose advanced materials for restoring, cleaning, and protecting works of art, but often no solutions are given for real-time refurbishing actions or aimed preventive conservation through the creation of buffer zones and controlled surrounding environments. Membranes can work as dynamic interfaces, enabling the exchange of mass, charge, and energy, along with other types of information, once targeted environmental conditions and the extent of variables are identified, combining the principles of long-lasting conservation and compatibility with that of reversibility. In this perspective, the chance to host responsive functions makes the membranes, with the support of process engineering, a real challenge in addressing a category of sensing devices that are easy to integrate with AIoT technology and, hence, suited to produce monitoring-reactive systems. This kind of practice is expected to pave the way for original, practical, and efficient remote-control actuating solutions, enabling one to check in a short time the ambient quality and readdress it to the conditions necessary to make the conservation effective and long-lasting.

Undoubtedly, membrane technology could provide new smart, lightweight, as well as noninvasive solutions within the context of heritage materials. Many of the solutions proposed herein do not necessitate that materials come into contact with the objects they are meant to protect. However, it may be necessary to produce films or coatings for in situ protection or cleaning procedures. In these cases, criteria of compatibility need to be even more carefully discerned, in addition to those of transparency and inertia. Today, great attention is paid to green and biodegradable materials, including natural polymers and plant-based agents, to form UV-blocking, antioxidant, and antibiofouling membranes. It is, however, relevant to observe that membrane durability depends heavily on the quality of the environment with which it comes into contact. Some materials, especially polymers, could be susceptible to chemical and physical aging. Mixed and self-healing matrixes are increasingly proposed to enhance membrane stability and counteract weakening events due to weathering or prolonged exposure to degrading sources. Also, the membrane performance depends a lot on fouling phenomena, and for this reason, the research orientation is toward the use of self-cleaning materials and those resistant to dirt adhesion.

A particular advantage concerning the use of membranes in cultural heritage contexts is the related modularity that allows for designing devices on different length scales, so that miniaturized as well as extensive structures can be realized with different configurations and without modifying the visual impact of the works of art. The cost-effective production of membranes is somewhat well-established on scale, including continuous manufacturing and surface functionalization. Today, the major challenge is rather to identify new chemical and eco-friendly formulations to combine with automated manufacturing processes, with the dual purpose of decreasing the environmental footprint and further reducing the membrane cost per m^2^. Although many issues are still open, the combination of membrane technology and cultural heritage conservation is envisaged as a long-term strategic approach.
